# Ability of routinely collected clinical factors to predict good visual results after primary Descemet membrane endothelial keratoplasty: a cohort study

**DOI:** 10.1186/s12886-022-02574-w

**Published:** 2022-08-24

**Authors:** Florian Bloch, Vincent Dinot, Christophe Goetz, Yinka Zevering, Louis Lhuillier, Jean-Marc Perone

**Affiliations:** 1 Department of Ophthalmology, Metz-Thionville Regional Hospital Center, Mercy Hospital, 1 Allée du Château, CS 45001, 57085, Metz-Cedex 03, France; 2 Clinical Research Support Unit, Metz-Thionville Regional Hospital Center, Mercy Hospital, Metz, France

**Keywords:** DMEK, Fuchs endothelial corneal dystrophy, Prognostic factors, Final postoperative BCVA, Patient age

## Abstract

**Background:**

A comprehensive analysis of routinely collected pre/perioperative demographic/clinical factors that could predict final visual acuity after primary Descemet membrane endothelial keratoplasty (DMEK) has not been conducted previously.

**Methods:**

A retrospective monocenter cohort study was performed with consecutive patients with Fuchs endothelial corneal dystrophy (FECD) who underwent DMEK or triple-DMEK (DMEK combined with cataract surgery) in 2016–2020 in a French tertiary-care hospital. DMEK-only patients were pseudophakic. Patients were followed for 12 months. Surgery was considered successful when 12-month best-corrected visual acuity (BCVA) was ≤0.1 logMAR (≥0.8). Exploratory multivariate analysis was conducted with the following routinely collected variables to determine their ability to predict 12-month BCVA: patient age and sex; graft donor age; triple DMEK; preoperative values of BCVA, endothelial cell density (ECD), central corneal thickness (CCT), and mean anterior keratometry; and rebubbling.

**Results:**

Of 100 eyes (100 patients; mean age, 72 years; 61% female), 81 achieved a 12-month BCVA of ≤0.1 logMAR. Logistic regression analysis showed that older age was a significant prognosticator for 12-month BCVA > 0.1 logMAR (Odds Ratio = 0.914, 95% confidence intervals = 0.846–0.987; *p* = 0.02).

**Conclusions:**

An older age associated with worse visual acuity outcomes after DMEK. This was confirmed by our analysis of the literature and supports the notion that DMEK should be conducted without delay once symptoms appear. Patient sex, donor age, triple-DMEK, and anterior keratometry also did not predict final BCVA in the literature. Preoperative CCT, ECD, and BCVA, and rebubbling occasionally appear in the literature as BCVA predictors, possibly reflecting an underlying ECD-BCVA axis.

## Background

The current standard surgical treatment for Fuchs endothelial corneal dystrophy (FECD) is Descemet membrane endothelial keratoplasty (DMEK), which was first described in 2006 by Melles et al. [1]. In DMEK, an endothelio-descemetic graft is used to replace the pathological endothelium and its Descemet membrane.

To date, a number of studies have searched for possible predictors of good visual acuity (VA) outcomes after DMEK for FECD [2–24]. All have some limitations: one included patients with corneas at both the early and end stages of FECD (i.e. the preoperative VAs ranged from very low to almost normal [5]); others examined variables that are not measured during the standard preoperative ophthalmological assessment before DMEK (anterior surface irregularities [3, 21] and anterior corneal haze [25, 26]); and many examined a mixture of indications rather than FECD only [5–8, 10–14, 16, 18–20, 22, 23], which are driven by different pathophysiologies [27–29] and can influence the final best corrected VA (BCVA) [12, 18, 20]. Most importantly, none systematically assessed a large array of routinely recorded pre/perioperative demographic and clinical factors for their ability to predict final BCVA after DMEK in FECD patients with explanatory multivariate analysis. All of these factors have the potential to shape final VA because they could influence the endothelial function of the graft, namely, its ability to pump out the excess fluid in the corneal stroma.

Therefore, the present retrospective cohort study was conducted to identify routinely collected demographic and clinical factors that predict good 12-month BCVA after DMEK for FECD. The results were compared to previously published results.

## Methods

### Study design and ethics

This retrospective monocenter observational study was conducted in the Department of Ophthalmology of the Metz-Thionville Regional Hospital Center, Lorraine University, Mercy Hospital (Metz, France). All patients gave verbal and written informed consent. The consent procedure was conducted in accordance with the reference methodology MR-004 of the National Commission for Information Technology and Liberties of France (No. 588909 v1). Ethics Committee approval was obtained from the French Society of Ophthalmology (Institutional Review Board (IRB) 00008855 Société Française d’Ophtalmologie IRB #1). The study was conducted according to the tenets of the Declaration of Helsinki and was registered at www.clinicaltrials.gov (No. NCT04469933, accessed July 14, 2020).

### Patient selection

The medical records were examined to identify all consecutive adult (≥18 years) patients who underwent primary DMEK between March 2016 and December 2020 to treat FECD characterized by cornea guttata with corneal edema (but no subepithelial scarring) that caused visual symptoms and reduced VA. Patients either underwent DMEK alone or triple DMEK (phacoemulsification followed immediately by DMEK). Patients who underwent DMEK alone were pseudophakic. Eyes were excluded if they had undergone prior eye surgery other than cataract surgery; had other indications for DMEK surgeries; had other diseases (i.e. corneal diseases other than FECD, important retinal or optic nerve diseases, and amblyopia); the DMEK surgery involved intraoperative complications (tearing or complicated graft unfolding), primary failure (defined as a cornea that remained edematous after the intervention), or secondary failure (defined by corneal decompensation after an initial period where the graft was functional); were lost to 12-month follow-up; or, in the case of bilateral DMEK, were the second operated eye.

### Graft preparation, surgical techniques, postoperative care, and follow-up

All corneal grafts were supplied by the tissue banks of Nancy or Besançon (France) and were kept in organoculture at 31 °C [30] in accordance with French bioethical laws. All had a requested endothelial cell density (ECD) greater than 2400 cells/mm^2^.

All procedures were performed by the same experienced surgeon (JMP). Inferior peripheral iridotomy with the Nd-YAG laser was performed during the preoperative check-up at least 15 days before the operation. Surgery was conducted under general anesthesia or, if the patient’s health status was precarious and contraindicated the use of general anesthesia, under peribulbar locoregional anesthesia (7 mL of ropivacaine 7.5 mg/mL).

All patients underwent DMEK as described [31]. Pseudophakic eyes underwent DMEK alone while phakic eyes underwent triple DMEK [32]. Triple DMEK involved phacoemulsification in micro-coaxial MICS mode (Stellaris PC, Bausch & Lomb, Rochester, New York, USA) using the subluxation technique [33] or the divide-and-conquer [34] technique followed by intraocular lens implantation in the posterior chamber with a flexible injectable implant (CT Asphina 409 M, Carl Zeiss Meditec AG, Jena, Germany). Careful aspiration of the viscoelastic (Healon GV®, Johnson & Johnson Vision, New Jersey, USA) and intracameral injection of 0.1 mL of Miochol-E® (acetylcholine chloride 20 mg/2 mL; Bausch & Lomb, Rochester, New York, USA) were performed at the end of the cataract procedure. Thereafter, DMEK was conducted with a 8 mm-diameter graft. A sterile air bubble was used for the tamponade at the end of surgery; its size was equivalent to 80% of the volume of the anterior chamber. If the epithelium seemed even slightly pathological, it was removed.

All patients were hospitalized for 3 days in the Ophthalmology Unit. They were followed up there or during outpatient visits on days 1, 7, and 15 and months 1, 3, 6, and 12.

If, during postoperative follow-up, more than a third of the graft had detached or graft detachment was threatening the visual axis [35, 36], a new tamponade was created under topical anesthesia (0.4% oxybuprocaine hydrochloride, Thea, France). This rebubbling procedure was repeated if necessary. Pilocarpine was not used during the early postoperative days.

Patients who developed cystoid macular edema and experienced a drop in VA were treated with oral acetazolamide (Diamox®, Sanofi, France; one 250 mg tablet 3 times/day for 1 month) and NSAID (indomethacin 0.1%, Chauvin, France; 4 times/day for 1 month).

Patients who presented with allograft rejection (defined as the presence of a line of retro-descemetic precipitates) were treated for 1 month with combined treatment composed of an anti-inflammatory corticosteroid ointment (Sterdex®, which contains dexamethasone with oxytetracycline, Thea, France; 2 times/day) and an anti-inflammatory corticosteroid eyedrop (Maxidrol®, which contains dexamethasone, neomycin, and polymyxin B; Alcon, Fort Worth, Texas; 12 times/day for 1 week then 8, 6, and 4 times/day for the second, third, and fourth week, respectively).

### Variables collected and tested for prognostic significance

The variables that were collected from the medical records were: patient age at the time of surgery; patient sex; graft donor age; graft origin; type of anesthesia (general or peribulbar); use of triple DMEK; BCVA before and 12 months after surgery (measured on the Monoyer scale and converted to a logMAR scale); graft ECD before (measured by the tissue bank) and 6 and 12 months after DMEK (measured by non-contact specular microscope CEM-530; Nidek Co. Ltd., Japan); central corneal thickness (CCT) before and 15 days and 12 months after DMEK (measured by anterior segment optical coherence tomography; RS-3000; Nidek Co. Ltd., Japan); mean anterior keratometry at baseline (measured by Visionix Luneau L67 auto-kerato-refractometer, France); postoperative development of cystoid macular edema or allograft rejection; and use of ≥1, one, or ≥ 2 rebubbling sessions for graft detachment.

The pre/perioperative variables were tested for prognostic significance, namely, patient age and sex, graft-donor age, triple DMEK, preoperative BCVA, ECD, CCT, and mean anterior keratometry values, and rebubbling.

DMEK alone and triple DMEK were considered to have yielded a good visual result if the BCVA at 12 postoperative months was ≤0.1 logMAR (≥0.8). This threshold has been used by others to signify successful DMEK surgery [6, 7, 20, 37, 38].

### Statistical analyses

Normality analysis with both the Shapiro-Wilk and Kolmogorov-Smirnov tests showed that of the continuous variables, graft donor age, BCVA, and CCT were not normally distributed. These data were expressed as median (interquartile range [IQR]). The normally distributed continuous variables were expressed as mean ± standard deviation. The remaining variables were expressed as *n* (%). The eyes that did and did not achieve a BCVA of ≤0.1 logMAR at 12 months were compared in terms of all variables by Student’s *t*-test, Wilcoxon test, or Chi-squared test, as appropriate. The variables were then subjected to multivariate analysis by using a logistic regression model. All variables were included in the multivariate model according to the explanatory modeling approach [39, 40]. The data were expressed as Odds Ratios (OR) and 95% confidence intervals (CI). A subanalysis to determine the correlation between preoperative and 12-month postoperative BCVA was conducted with Spearman test. Two additional subanalyses assessing the difference between eyes that did and did not achieve a BCVA of ≤0.1 logMAR at 12 months in terms of 6- or 12-month postoperative ECD and 6- or 12-month postoperative CCT were conducted with Student’s *t*-test and Wilcoxon test, respectively. The significance threshold was set at 5%. All statistical analyses were conducted by using SAS software (version 9.3, SAS Inst., Cary, NC, USA).

## Results

**Fig. 1:**
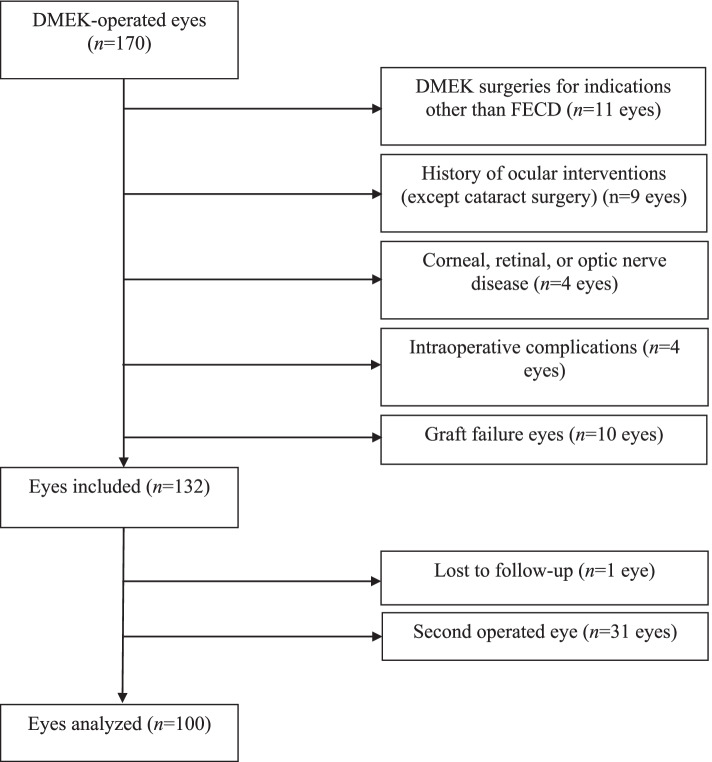
Flow chart showing the disposition of the operated eyes during the study. DMEK, Descemet membrane endothelial keratoplasty; FECD, Fuchs endothelial corneal dystrophy

Chart review identified 170 eyes that underwent DMEK during the study period. Of these, 70 eyes were excluded because they had undergone DMEK surgery for indications other than FECD (*n* = 11), had undergone prior eye surgery other than cataract surgery (*n* = 9), had another disease (*n* = 4), the DMEK surgery involved intraoperative complications (n = 4), there was primary/secondary graft failure (*n* = 10), the patient was lost to follow-up (n = 1), or the eye was the second operated eye in bilateral DMEK (*n* = 31). Thus, 100 eyes were included in the study (Fig. 1).

### Patient and eye characteristics

Table 1 summarizes the demographic, surgical, and pre/peri- and postoperative clinical characteristics of the 100 patients and eyes. The average age of the patients was 72 years and 61% were women. The median age of the graft donors was 75 years. In terms of surgery, the graft originated from Besançon and Nancy in 55 and 45% of cases, respectively, 97% received general anesthetic, and 43% underwent triple DMEK.

**Table 1:** Demographic, surgical, and clinical characteristics of 100 eyes that underwent primary DMEK for FECD

Characteristics	Mean ± SD, median (IQR), or *n* (%)
Patient age, years	72 ± 9
Female sex	61 (61)
Graft donor age, years	75 (70–82)
Origin of the graft, city	
Besançon	55 (55)
Nancy	45 (45)
Anesthesia	
General	97 (97)
Peribulbar	3 (3)
Triple DMEK	43 (43)
Preoperative BCVA, logMAR	0.7 (0.5–0.7)
M12 postoperative BCVA, logMAR	0.05 (0–0.1)
M12 BCVA ≤0.1logMAR	81 (81)
Preoperative (graft) ECD, cells/mm^2^	2538 ± 207
M6 postoperative ECD, cells/mm^2^	1318 ± 431
M12 postoperative ECD, cells/mm^2^	1165 ± 402
Preoperative CCT, μm	620 (587–660)
Postoperative D15 CCT, μm	600 (550–654)
Postoperative M12 CCT, μm	531 (512–559)
Mean anterior keratometry, D	43.7 ± 1.5
Cystoid macular edema	4 (4)
Allograft rejection	1 (1)
One or more rebubbling sessions	30 (30)
Only one rebubbling session	21 (21)
≥2 rebubbling sessions	9 (9)

*BCVA* best corrected visual acuity, *CCT* central corneal thickness, *DMEK* Descemet membrane endothelial keratoplasty, *D15* postoperative day 15, *ECD* endothelial cell density, *M6* month 6, *M12* month 12, *IQR* interquartile range

Before DMEK, the median BCVA was 0.7 logMAR; at 12 months, it was 0.05 logMAR and 81% of the patients had a BCVA ≤0.1 logMAR. Mean preoperative (graft) ECD was 2538 cells/mm^2^. This changed to 1318 and 1165 cells/mm^2^ at 6 and 12 months, respectively (mean loss of 48 and 54%, respectively). This reflects the fact that learning curve patients were included in the study. Median preoperative CCT was 620 μm. This changed to 600 and 531 μm at Day 15 and 12 months, respectively. Preoperative mean anterior keratometry was 43.7 D.

After surgery, 4% of eyes presented with a drop in VA due to cystoid macular edema, 1% rejected the allograft, and 30% required at least one rebubbling session. Most (21/30, 70%) only required one session; the remaining nine cases needed at least two rebubbling sessions.

### Prognostic factors for good DMEK outcome

Table 2 shows the factors that predicted a good DMEK outcome (defined as 12-month BCVA ≤0.1 logMAR). Univariate analyses showed that younger patients had better DMEK outcomes than older patients (70 vs. 77 years; *p* = 0.001; Student’s t-test). Age was also a significant prognosticator of a good DMEK outcome on explanatory multivariate analysis (Odds Ratio = 0.914, 95% confidence intervals = 0.846–0.987; *p* = 0.02). The effect was relatively weak: an Odds Ratio of 0.91 means that the chance of achieving an final BCVA of < 0.1 logMAR decreases by a few percent for every year of age. None of the other factors that were studied associated with better DMEK outcomes (Table 2).

**Table 2:** Association between prognostic factors and BCVA of ≤0.1 LogMAR at 12 postoperative months

Factors	M12 BCVA ≤0.1 logMAR (*n* = 81)	M12 BCVA > 0.1 logMAR (*n* = 19)	Univariate^a^ p	Multivariate^b^	
				OR (95% CI)	*p*
Patient age, years	70 ± 9	77 ± 7	0.001	0.914 (0.846; 0.987)	0.02
Female sex	40 (59)	13 (68)	0.60	1.264 (0.386; 4.134)	0.70
Graft donor age, years	75 (70–82)	74 (56–85)	0.68	1.023 (0.980; 1.068)	0.31
Triple DMEK	38 (47)	5 (26)	0.13	2.875 (0.802; 10.304)	0.10
Preop BCVA, logMAR	0.5 (0.5–0.7)	0.7 (0.7–0.7)	0.25	0.922 (0.139; 6.130)	0.93
Preop ECD, cells/mm^2^	2535 ± 207	2549 ± 211	0.79	1.001 (0.998; 1.004)	0.46
Preop CCT, μm	620 (589–662)	620 (579–654)	0.64	1.003 (0.993; 1.013)	0.53
Mean ant. Keratometry, D	43.7 ± 1.5	43.4 ± 1.5	0.36	1.368 (0.887; 2.110)	0.16
One or more rebubbling	24 (29)	6 (32)	0.99	1.152 (0.341; 3.898)	0.82

The data are presented as mean ± standard deviation, median (interquartile range), or *n* (%), as appropriate

Akaike Information Criterion = 99.245

*ant.* Anterior, *BCVA* best spectacle-corrected visual acuity, *CI* confidence interval, *CCT* central corneal thickness, *DMEK* Descemet membrane endothelial keratoplasty, *ECD* endothelial cell density, *M12* Month 12, *OR* Odds Ratio

^a^Student’s *t*-test, Wilcoxon, or Chi-squared test, as appropriate

^b^Logistic regression

### Subanalysis of the correlation between preoperative and postoperative BCVA

A weak positive correlation was observed between preoperative and 12-month BCVA (*r* = 0.19, *p* = 0.06).

### Subanalysis of the relationship between postoperative CCT and final BCVA

We observed that compared to eyes with poor BCVA outcomes, eyes with good BCVA outcomes tended to have smaller CCTs at 6 (median [IQR] = 526 [510–553] vs. 533 [522–580] microns; *p* = 0.13) and 12 postoperative months (527 [510–557] vs. 551 [530–564] microns; *p* = 0.09).

### Subanalysis of the relationship between postoperative ECD and final BCVA

Compared to eyes with poor final BCVA, eyes with good BCVA outcomes had significantly higher ECD at 6 (1364 ± 424 vs. 1121 ± 417 cells/mm^2;^ *p* = 0.03) and 12 postoperative months (1218 ± 395 vs. 940 ± 361 cells/mm^2^; *p* = 0.01).

## Discussion

This cohort study assessed nine routinely collected pre/perioperative factors for their ability to predict 12-month BCVA after DMEK for FECD. The cohort largely resembled the cohorts of other studies on consecutive patients who underwent DMEK for FECD with tissue-bank transplants in terms of demographics, pre/perioperative and postoperative variables, complication rates [11, 37, 41–43], and final BCVA: previously reported 12-month BCVA ranges from 0.2 to − 0.1 logMAR [3–5, 9], which is similar to the present study’s finding (0.05 logMAR). In the present study, only age was a significant prognosticator of good DMEK outcomes. The 24 previous studies searching for variables that predict final VA are summarized in Table 3 [2, 3, 14, 17–20]. Most examined cohorts with mixed indications for DMEK, final VA was mostly recorded within 12 months of surgery, nine conducted multivariate analyses, and most only assessed one or two of the nine pre/perioperative variables that we examined. Below, we first discuss the findings regarding the significant prognosticator (age). The other factors that did not serve as prognosticators in our study were then also reviewed.

**Table 3:** Summary of other studies examining whether the pre/perioperative factors we looked at associate with final visual acuity after DMEK

Author year	No. eyes	Indic.	Postop time point	Type analysis	Patient age	Patient sex	Donor age	Triple DMEK	Preop BCVA	Preop ECD	Preop CCT	Mean anter. Kerat.	Rebubb.
Chauras. 2014 [2]	492	FECD	6 mo	Uni/Multi.	< 0.0001^a^			< 0.0001^b^					
Van Dijk 2014 [3]	118	FECD	6 mo	Multi.	< 0.001	NS		NS	< 0.001				
Schaub 2016 [14]	529	mixed	12 mo	Univ.			NS						
Ham 2016 [18]	250	mixed	6 mo	Multiv.	0.0016								
Schlögl 2016 [19]	97	mixed	3 y	Univ.				NS					
Peraza 2017 [20]	396	mixed	2 y	Multi.	0.0012	NS				NS	< 0.0001		0.0003
Schaub 2017 [21]	160	FECD	2 y	Univ.						NS	NS		
Neiter 2017 [22]	19	mixed	6 mo	Univ.							NS		
Gerber 2017 [23]	760	mixed	6 mo	Univ.									NS
Mechels 2017 [24]	94	FECD	3 mo	Univ.									NS
Brockm. 2019 [4]	108	FECD	12 mo	Univ.					NS	NS	0.014		
Schaub 2019 [5]	1748	mixed	12 mo	Univ.			NS						
Schritt. 2019 [6]	1084	mixed	12 mo	Univ.					0.011		NS		
Godin 2019 [7]	136	mixed	UC	Univ.									NS
Singh 2019 [8]	50	mixed	3 mo	Univ.			0.03	NS					
Shah. 2020 [9]	114	FECD	12 mo	Univ.				NS					
Birbal 2020 [10]	738	mixed	6 mo	Multi.				NS					
Birbal 2020 [11]	393	mixed	5 y	Multi.	NS	NS		NS					< 0.0001
Besek 2022 [12]	150	mixed	7 y	Multi.	0.009		NS	NS					NS
Gund 2021 [13]	463	mixed	12 mo	Multi.									NS^c^
Agha 2021 [15]	33	FECD	9 mo	C-C									0.03
Romano 2022 [16]	91	mixed	12 mo	Univ.				NS					NS
Moskwa 2022 [17]	111	FECD	12 mo	Univ.							NS		
Our study	100	FECD	12 mo	Multi.	0.02	NS	NS	NS	NS	NS	NS	NS	NS

*anter.* Anterior, *C-C* case control study, *CCT* central corneal thickness, *DMEK* Descemet membrane endothelial keratoplasty, *ECD* endotehlial cell density, *FECD* Fuchs endothelial corneal dystrophy, *Indic.* indications, *mo* months, *kerat.* Keratometry, *Multiv.* multivariate, *NS* not significant, *Rebubb.* rebubbling, *UC* unclear, *Univ.* univariate, *y* year

^a^Multivariate analysis

^b^Univariate analysis

^c^Significant on univariate analysis but not after controlling for donor age, indication, graft cultivation time, and preoperative visual acuity

### Patient age

Six studies assessed the relationship between patient age and final VA after DMEK. All were multivariate analyses. Like us, four observed that an older age predicted worse final BCVA [2, 3, 18, 20]. However, one found no association [11] and another reported that a younger age predicted worse final VA [12]. The importance of age was noted by two studies as moderate and small [3, 20]. Since all studies were quite similar in terms of the independent variables that were included, the discrepancies between them may reflect the duration since DMEK was performed. Specifically, the five studies (including ours) that showed associations with older age examined cases 6–24 months after DMEK [2, 3, 18, 20], whereas the study that failed to find an association involved 5-year post-DMEK patients [11], and the study showing an association with younger age focused on patients 7 years after DMEK [12]. The latter two studies may be complicated by selection bias due to the tendency of geriatric patients to have a poorer BCVA in general than younger patients and to be lost to follow-up more often.

Notably, a multivariate analysis on patients who underwent Descemet stripping automated endothelial keratoplasty (DSAEK) or DMEK also observed that an older age associated with worse final VA [44].

Thus, an older age may limit the visual outcomes after DMEK, although the effect size may be small-moderate. Since we excluded patients with other ocular diseases, it is likely that this effect reflects an inherent frailty that is due to aging of the recipient cornea (e.g. loss of photoreceptors [45, 46] or neural adaptability [25]) rather than ocular comorbidity. Thus, it may be prudent to perform DMEK as soon as symptoms arise [25]. This is supported by the finding that very old patients (> 85–90 years) have more complications and smaller VA gains than younger patients [47, 48] and the fact that endothelial dystrophy progression generates irreversible corneal changes [6, 49, 50].

### Preoperative factors that did not have prognostic value in our study

#### Patient sex

Three other studies (all multivariate analyses) also failed to detect an association between patient sex and final VA [3, 11, 20].

#### Donor age

Three of four studies on donor age also did not find an association with final VA [5, 12, 14]. One was also a multivariate analysis [12]. The fourth study examined the first 50 DMEK patients in a facility and showed that older donor age (> 50 years) associated with better 3-month BCVA [8]. This may reflect the inexperience of the operator and the fact that older DMEK grafts scroll less tightly and are therefore easier to manipulate than younger grafts [51, 52]. Thus, graft age does not appear to influence the VA outcomes of DMEK. This is supported by a recent study showing that grafts from very old donors (≥80 years) have good VA results [53].

#### Triple DMEK

Of nine studies on triple DMEK, eight (including three multivariate analyses) also found no association between this variable and final BCVA [3, 8–12, 16, 19]. Thus, triple DMEK may not differ from pseudophakic DMEK in terms of final VA.

#### Preoperative BCVA

Two of three studies found an association between preoperative and postoperative BCVA [3, 4, 6]. One, a multivariate analysis, observed that preoperative BCVA was of moderate importance in predicting final BCVA [3]. The other, a univariate analysis, found linear relationships between preoperative and postoperative BCVA and reported that eyes with preoperative BCVA < 20/100 recover less well and more slowly than eyes with better BCVA [6]. The third study, a univariate analysis, did not observe a correlation between preoperative and postoperative BCVA [4]. Our present study also did not find an association but we did not directly compare these two variables; rather, the relationship between preoperative VA and two categories of 12-month VA (12-month BCVA of ≤0.1 or > 0.1 logMAR, respectively) was assessed. Notably, we observed a weak positive correlation between preoperative and 12-month postoperative BCVA. Preoperative BCVA may not have emerged as a prognosticator in our study because many exclusion criteria were used to select the patients, thereby resulting in a fairly homogeneous sample with relatively low baseline VA variation. This may have made it more difficult to observe an effect of preoperative BCVA.

Thus, preoperative BCVA may be weakly predictive of final BCVA. This may reflect endothelial dystrophy-induced morphological changes in the cornea that cause it to recover less well after DMEK [6].

#### Preoperative (graft) ECD

Three studies (one multivariate) also did not observe a relationship between preoperative (graft) ECD and final VA [4, 20, 21]. This is consistent with the fact that graft ECD only has obvious effects on postoperative VA if it drops below 1000 cells/mm^2^ after surgery [54, 55]. Consequently, corneal surgeons routinely request DMEK grafts with ample ECD reserve (generally > 2400 cells/mm^2^) [56].

Interestingly, our subanalysis showed that poor postoperative VA associated with significantly worse postoperative ECD. We speculate that postoperative, but not preoperative, graft ECD associates with postoperative BCVA because preoperative ECD measurements are distorted by preoperative factors (e.g. time between counting and surgery [57, 58]) that disappear after surgery, thus allowing postoperative ECD to better reflect the intrinsic healthiness of the graft endothelium and its ability to clear the corneal stroma.

Thus, while preoperative (graft) ECD may not predict final VA after DMEK, postoperative ECD could. Since this is a post-surgical measurement, it has no clinical utility but it does suggest that final VA also depends on ECD at densities above the decompensation threshold of 1000 cells/mm^2^.

#### Preoperative CCT

Four of six studies also failed to find relationships between preoperative CCT and postoperative BCVA. All were univariate analyses [6, 17, 21, 22]. One was by our group: it showed that preoperative CCT did not correlate with BCVA at various postoperative timepoints [17]. By contrast, a multivariate analysis observed that higher preoperative CCT predicted final BCVA, albeit with a weak clinical effect size [20], and a univariate analysis found that < 625-μm preoperative CCTs associated with better 12-month BCVA than thicker preoperative CCTs [4]. Our previous study was unable to replicate the latter finding [17].

Interestingly, our third subanalysis in the present study showed that poor BCVA outcomes tended to associate with thicker postoperative CCTs. This is supported by our previous study, which observed that postoperative CCT correlated with postoperative BCVA [17]. We speculate that postoperative CCT may associate with postoperative VA because it accurately reflects how well the corneal (graft) endothelium pumps the excess fluid out of the host cornea; by contrast, preoperative (host) CCT measurements may be blunted by preoperative factors that disappear after DMEK. Similarly, our recent study on DSAEK showed that postoperative, but not preoperative, central graft thickness predicted postoperative BCVA [59]. Thus, postoperative ECD and CCT, but not their preoperative counterparts, may associate with postoperative VA because they better reflect the intrinsic healthiness of the graft and its ability to reduce corneal edema.

#### Rebubbling

Four of nine studies on rebubbling were multivariate analyses that reported different outcomes despite being conducted similarly: in two, mild and/or severe detachment predicted worse final VA [11, 20] whereas in another two, neither mild/severe detachment nor rebubbling predicted VA [12, 13]. Why these studies differed is unclear. Of the remaining five (univariate) studies, only one found that rebubbling associated with worse final VA [7, 15, 16, 23, 24].

Thus, it remains unclear whether rebubbling affects final VA. Since a multivariate analysis of 841 eyes [60] and a large prospective registry study on 752 eyes [61] observed that rebubbling associates with greater endothelial cell loss, and our analysis above suggests that postoperative ECD may shape final VA, it may be that some studies but not others are able to detect the rebubbling-ECD-BCVA relationship due to their patient composition and/or their peri/postoperative practices.

#### Mean anterior keratometry

To our knowledge, the ability of anterior keratometry to predict final BCVA after DMEK has not been reported previously. We did not find that it has any predictive value. Theoretically, if there is a rebubbling-ECD-BCVA relationship (as proposed above), it is possible that corneal curvature could affect final VA because DMEK grafts may adhere less stably to flatter corneas and require rebubbling. This notion is supported by Romano et al., who suggested that rebubbling occurs more often in DMEK grafts that are less adhesive due to longer culture times [16]. However, posterior keratometry, which directly measures posterior curvature, may be more likely to show a relationship between corneal curvature and postoperative BVCA. We did not assess postoperative keratometry in the present study because it is a time-consuming procedure and the study aimed to identify simple, routinely collected markers of visual outcome after DMEK.

### Study limitations

The present study has several limitations. First, our sample size was relatively small. Thus, our failure to detect an association between final VA and a pre/perioperative variable does not necessarily mean that it does not exist. However, our findings are largely borne out by other studies, many of which had much higher sample sizes (Table 3). Second, our study was retrospective and single-center, which could lend itself to information and selection bias. However, the medical record database was prospectively maintained. Third, because the study aimed to identify quantifiable, comparable, and reproducible prognostic criteria for long-term DMEK outcomes, stringent exclusion criteria were applied to eliminate potential patient- and surgery-related confounders. However, this could have led to selection bias that resulted in a cohort that does not fully resemble the populations that ophthalmological surgeons see on a regular basis. Nevertheless, as indicated above, our cohort did not differ markedly from other cohorts in terms of demographic and clinical characteristics and final BCVA. Fourth, the BCVA> 0.1 logMAR group was small, which may have reduced study power, therefore causing some potential prognostic factors to fail to achieve significance. Fifth, we did not study the prognostic capacity of graft-storage duration, which may influence postoperative ECD loss [58]. Finally, we also did not study the relationship between final BCVA and cardiovascular pathology, particularly diabetes, which seems to associate with greater ECD loss [62, 63]. This is also true for insulin-treated diabetes, which also associates with greater graft detachment and rebubbling rates [63].

## Conclusions

Our study showed that an older age is a prognostic factor for worse 12-month BCVA after DMEK. This was confirmed by other studies in the literature and supports the notion that DMEK should be performed without delay when symptoms arise. Our study and the literature also suggest that patient sex, donor age, triple DMEK, and anterior keratometry are not significant prognosticators of VA after DMEK. However, preoperative VA may weakly predict final VA. Moreover, while preoperative ECD and CCT and rebubbling may not play strong roles as VA predictors, they are occasionally detected as predictors in the literature, possibly because of an underlying ECD-VA axis that can be detected in specific cohort and peri/postoperative settings.

## Data Availability

The datasets used and analyzed during the current study are not publicly available due to patient protections and institutional policy but are available from the corresponding author on reasonable request.

## Abbreviations

BCVA: Best corrected visual acuity;
CCT: Central corneal thickness
DMEK: Descemet membrane endothelial keratoplasty
DSAEK: Descemet stripping automated endothelial keratoplasty
ECD: Endothelial cell density
FECD: Fuchs endothelial corneal dystrophy
VA: Visual acuity
